# Clinical efficacy and kinematic analysis of Chinese knotting technique-assisted posterior cruciate ligament reconstruction: A retrospective analysis

**DOI:** 10.1097/MD.0000000000037840

**Published:** 2024-04-26

**Authors:** Bohan Xiong, Yang Yu, Bing Xie, Guoliang Wang, Xianguang Yang, Jinrui Liu, Ziming Gu, Yanlin Li

**Affiliations:** a Department of Sports Medicine, First Affiliated Hospital of Kunming Medical University, Kunming, Yunnan, China.

**Keywords:** Chinese knotting technique, gait analysis, kinematics of knee joint, knee score, posterior cruciate ligament reconstruction, secondary microscopic examination

## Abstract

To investigate the clinical efficacy and knee joint kinematic changes of posterior cruciate ligament (PCL) reconstruction assisted by Chinese knotting technique (CKT). A retrospective analysis was conducted on 88 cases of PCL reconstructive surgery admitted between September 2016 and September 2020. All patients were operated on by the same senior doctor and his team. The patients were divided into 2 groups according to whether the CKT was applied, with 44 cases in each group. Both groups received active rehabilitation treatment after surgery. All patients were followed up for more than 2 years. International knee documentation committee, hospital for special surgery (HSS), and Lysholm scores were used to evaluate the clinical efficacy of the 2 methods at 3, 12, and 24 months after surgery. The motion cycle and kinematic indices of the knee joint were measured by the Opti_Knee three-dimensional motion measurement system before surgery and at 3, 12, and 24 months after surgery. A secondary arthroscopic examination was performed at 12 months after surgery, MAS score was used to evaluate the secondary endoscopic examination of PCL. All the patients had wound healing in stage I without infection. International Knee in both sets Documentation Committee scores, HSS scores and Lysholm scores were gradually improved at all time points (*P* < .05); compared with the traditional group, the HSS score was higher in the reduction group 12 months after surgery (*P* < .05), but there was no significant difference at 24 months after surgery. 12 months and 24 months after 3 dimensional motion measurement system using Opti_Knee showed a reduction group before and after displacement and displacement of upper and lower range than the traditional group (*P* < 0. 05). One year after surgery, the good and good rate of MAS score reduction group was higher than traditional group. CKT assisted PCL reconstruction can improve the subjective function score of the affected knee joint and the results of secondary microscopy. Satisfactory knee kinematic function can be obtained in the early stage, and the anteroposteric relaxation of the knee joint can be reduced.

## 1. Introduction

Arthroscopic reconstruction of posterior cruciate ligament (PCL) has become the most effective and common treatment for PCL fractures due to its advantages of minimal trauma and quick recovery. To date, tendon grafts after PCL reconstruction have mainly left with different degrees of relaxation.^[1–3]^ After ligament reconstruction, the overall mechanical strength of tendon graft may decrease due to the disintegration of original collagen fibers, and radical rehabilitation exercises may cause graft relaxation and refracture.^[4]^ In recent years, scholars have used FiberTape^TM^, InternalBrace^TM^, Ethibond^TM^, and other high-strength sutures (bands) to perform Chinese knotting technique (CKT) to assist ACL reconstruction, and they achieved satisfactory biomechanical histological, and clinical results in vivo and in vitro.^[5–7]^ In our previous research, 2#Ethibond^TM^ wires were independently innovated using “triangle braiding technology” to weave tension-bracing wires with an appropriate elastic modulus, and autologous hamstring tendon transplantation was utilized to reconstruct ACL, in order to significantly improve clinical efficacy and knee kinematics.^[8]^ Therefore, in the present study, CKT was applied to PCL reconstruction, and the clinical efficacy and knee kinematics of CKT-assisted PCL reconstruction were discussed, hoping to achieve the same satisfactory effect as CKT-assisted ACL reconstruction. This study analyzed the clinical data of 88 patients with PCL rupture who were admitted to Department of Sports Medicine, the First Affiliated Hospital of Kunming Medical University (Kunming, China) between September 2016 and September 2020. The clinical efficacy of CKT-assisted PCL reconstruction was evaluated by subjective quantitative score and secondary microscopic examination results, and the knee joint kinematic data were collected and analyzed.

## 2. Research object and method

### 2.1. Inclusion and exclusion criteria

#### 2.1.1. Inclusion criteria

① Male or female patients who aged from 18 to 55 years old; ② patients with a clear history of knee injury, who were in a satisfactory health status before the injury, without a history of chronic knee injury, and were able to perform routine exercises; ③ after physical examination, drawer test was positive, reverse Lachman test was positive, and step sign was positive; ④ magnetic resonance imaging (MRI) showed PCL fracture; ⑤ PCL fracture was confirmed by arthroscopy, and meniscus and articular cartilage injuries were moderate or mild or nondestructive.

#### 2.1.2. Exclusion criteria

① Complication of knee joint with rheumatoid arthritis, infections, tumors, and tumor-like lesions; ② the existence of multiple ligament injuries or fractures; ③ injury was evaluated as severe articular cartilage injury or meniscus injury; ④ refusal to participate in the clinical trial and missing interviewees.

All patients and their family members gave informed consent to this study and signed an informed consent form, which was approved by the First Affiliated Hospital of Kunming Medical University Ethics Committee.

### 2.2. Research subjects and groups

A total of 88 patients who underwent arthroscopic PCL reconstruction in the Department of Sports Medicine, the First Affiliated Hospital of Kunming Medical University between September 2016 and September 2020 due to PCL rupture were randomly divided into 2 groups according to different treatment methods. In the CKT group, 44 patients were treated with internal bracing-assisted autologous hamstring tendon transplantation for arthroscopic single-bundle reconstruction of PCL. In the traditional group, 44 patients were treated with autogenous hamstring tendon grafts and single-bundle reconstruction of PCL under an arthroscope. There was no significant difference in the preoperative general data of PCL rupture patients between the CKT group and the traditional group, such as gender, age, injury side, body mass index (BMI), traumatic complications, and time from injury to surgery (Table 1).

**Table 1:** Comparison of patients’ preoperative general data between CKT group and traditional group (x̄ ± *s*).

Group	Gender		Age (*x̄ ±s*)	Side of injury		BMI (kg/m^2^, x̄±s)	Combined injury		Injury to the time of surgery (mouth, x̄±s)
	Male	Female		Left	Right		Have	Not	
CKT	30	14	39.0 ± 10.4	22	22	24.4 ± 2.3	24	20	2.6 ± 5.6
Traditional	35	9	39.6 ± 11.4	23	21	24.6 ± 2.3	23	21	2.6 ± 4.4
*t* value/χ^2^value	1.472		0.224	0.045		0.531	0.046		-0.011
*P* value	.225		.823	.831		.596	.831		.992

*Note*: The *t* value represented the independent-sample *t*-test of age, BMI, and time from injury to surgery between the CLT group and the traditional group.

χ^2^ represented the Chi-square test results of gender, injury side, and combined injury between the CKT group and the traditional group.

There was no significant difference in preoperative general data between the 2 groups (*P* > .05), indicating comparability of data.

### 2.3. Surgical methods

All patients were managed by a medical team led by a senior physician. Arthroscopic PCL reconstruction of knee joint was performed under combined epidural and lumbar anesthesia. The patient was placed in the supine position, and the tourniquet pressure was adjusted to 45 to 50 mm Hg after routine disinfection, towel placement, and blood displacement. Standard anterior medial and anterolateral approach incisions were made on the affected knee, both 1.0 cm in length, and PCL rupture was confirmed under a microscope. A 3.0 cm oblique incision was made on the medial side of the tibial tubercle, and the femoral thin tendon and semitendinosus tendon were removed as grafts to reconstruct the PCL.

**Figure 1.**
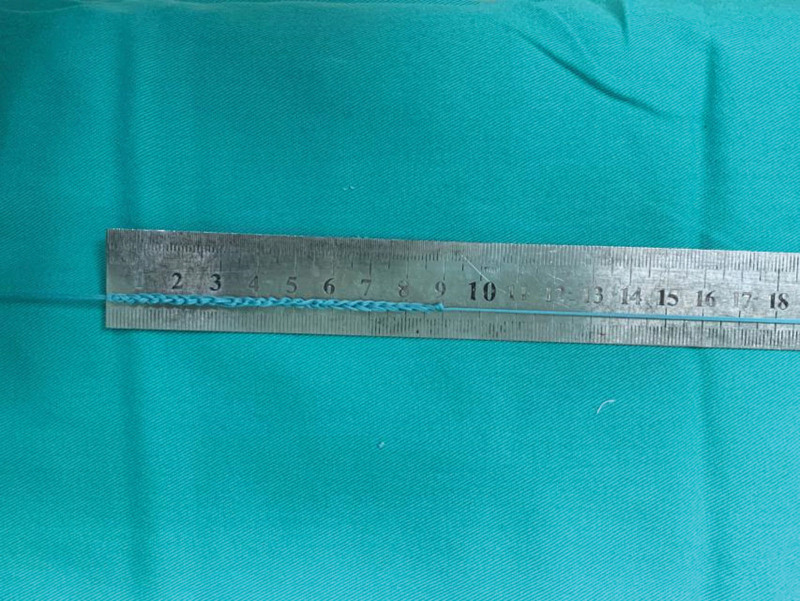
Two No. 2 Ethibond^TM^ wires were braided into tension reduction wires by the “triangle braiding technology.”

**Figure 2.**
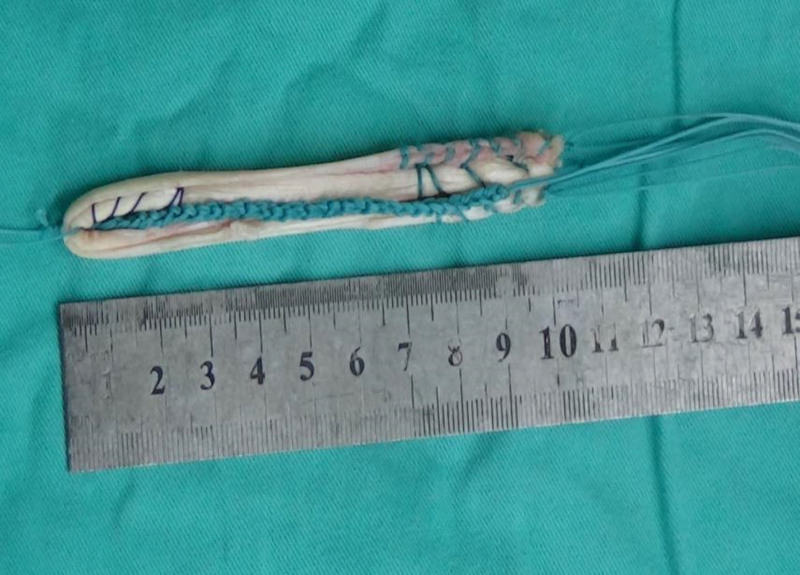
Folding the braided gracilis muscle and semitendinosus muscle in half into 4 strands and wrapping the tensioned line in the center.

**Figure 3.**
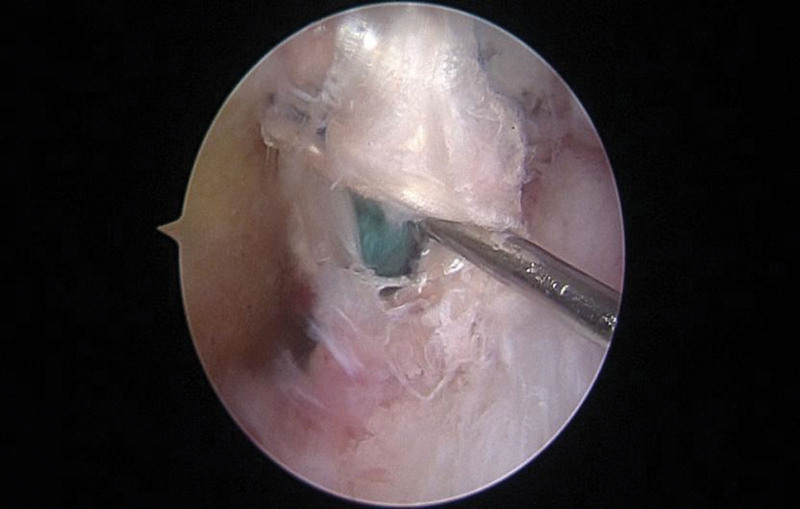
Arthroscopic observation of the PCL with internal reduction reconstruction, and the hook shows the reduction line located in the center of the graft tendon.

In the CKT group, thin femoris tendon and semitendinosus tendon were taken, and both ends of No. 2 Ethibond^TM^ conventional braided tendon were used. In addition, 2 No. 2 Ethibond^TM^ wires were woven into the bracing reduction wires using the independent innovation of “triangle weaving” method^[8]^ (Fig. 1). One end of the bracing line was knotted and fixed on the loop with a titanium plate with loop. The braided gracilis muscle and semitendinosus muscle were folded in half into 4 strands, and the bracing line was wrapped around and used for reconstruction with 80 N pre-stretch backup PCL (Fig. 2). In the traditional group, thin femoral tendon and semitendinosus tendon were taken, and both ends of the No. 2 Ethibond^TM^ conventional braided tendon were used. The braided gracilis muscle and semitendinosus muscle were folded in half into 4 strands without the use of bracing lines, and the 80N pre-stretch reserve PCL was used for reconstruction. The intercondylar fossa of the knee was cleaned, and the PCL femoral footprint stump was exposed and retained for reserve. Meniscus injury repair or partial excision was performed for patients with meniscus injury. The standard posteromedial and posterolateral approaches of the affected knee were both 1.0 cm long incisions, the posterior compartment was cleaned, the posterior mediastinum was opened, and the tibial footprint stump of PCL was exposed and retained for reserve. Femoral and tibial bone tunnels were made in the footprint area of PCL according to the length and thickness of the woven tendon. A titanium plate with loop was used for fixation of the femoral end (Smith & Nephew, London, UK), and hydroxyapatite interfacial screw was used for fixation of the tibial end (Smith & Nephew, London). The self-made “door-type nail” was used to strengthen the fixation below the outer entrance of the tibial tunnel.^[8]^ The tension and position of the reduced tension reconstructed PCL were assessed by arthroscopy again (Fig. 3).

### 2.4. Rehabilitation

Patients in the 2 groups were treated with local ice for 20 min/time, and 3 times a day, for 72 hours. At rest, the affected limb was cushioned from the middle and upper 1/3 of the calf to the distal end, and the knee was extended. A chuck support was used during training and was strictly worn for 3 months. Postoperative routine “straighten, bend, lift leg, contract muscle,” ankle pump exercise, straight leg lift (side lift, back lift), patella loosening exercise, and walking without losing weight were performed. The difference was mainly attributed to the bending angle of the affected knee and the weight bearing condition at different time points. Patients in the CKT group underwent rehabilitation exercise according to the aggressive rehabilitation program, while patients in the traditional group underwent rehabilitation exercise according to the conventional program (Table 2).

**Table 2:** Postoperative rehabilitation programs in the 2 groups.

	Post surgery duration(week)	1	2	3	4	5	6	7	8	9	10		11	12
CKT group	Angle of bending	0° to 70° passive buckling exercises			90°	100°	110°	120°	>120 degrees – full angle					
	The load	No weight bearing, straighten position to feel the stress			30%	50%	80%	100%	Full load bearing			Gradually do balance, strength, endurance, reflexes, proprioception and resistance training		
Traditional group	Angle of bending	0–30° passive buckling exercises			45° to 90°, up to 90° after 6 weeks			90° to 120°, up to 120° after 12 weeks						
	The load	No weight bearing					Lifting weight on crutches (30% to 60% of body weight)		Partial weight bearing (> 60% of body weight)			Full load bearing		

### 2.5. Observe indicators

International knee documentation committee (IKDC), hospital for special surgery (HSS), and Lysholm knee scores were used to evaluate the clinical efficacy of the 2 groups at 3, 12, and 24 months after surgery.^[9]^ In addition, the Opti_Knee three-dimensional motion analysis system (Shanghai Yidong Medical Technology Co., Ltd., Shanghai, China) was used to record 6 degrees of freedom (flexion and extension angles, internal and external pronation angles, internal and external rotational angles, front and back displacements, up and down displacements, and internal and external displacements) of the knee (the difference between the maximum and minimum values) at 3, 12, and 24 months after surgery, so as to evaluate the results of knee kinematic analysis.^[10–15]^ A secondary arthroscopic examination was performed at 12 months after surgery, and MAS score was calculated to evaluate the results of the secondary endoscopic examination.^[16]^

### 2.6. Statistical analysis

SPSS 17.0 software (IBM, Armonk, NY) was used to perform statistical analysis. Count data were compared by Chi-square test. Normally distributed measurement data, as confirmed by Kolmogorov–Smirnov (K–S) test, were expressed as mean ± standard deviation (*x* ± *s*), compared with paired t-test, multiple comparison analysis was carried out using Student-Newman-Keuls (SNK) method, and α level was set to 0.05. *P* < .05 was considered statistically significant. A power analysis demanded that 88 patients in each group to produce a power of 90% and a *P* value of .05.

## 3. Results

All patients in the 2 groups were recovered well after surgery, the incision was healed in stage I without infection, and there were no complications, such as stiffness, infection, nerve injury, and deep vein thrombosis after surgery. In addition, X-ray, computed tomography (CT), and MRI at 3 months after surgery indicated that the ligament and bone tunnel were well healed, and no internal fixation device was loosened. All patients were followed up for 24 months after surgery.

The preoperative and postoperative IKDC, HSS, and Lysholm scores in the 2 groups showed that the HSS scores in the CKT group were higher than those in the traditional group at 12 months after surgery (*P* < .05), while there was no significant difference in HSS scores between the 2 groups at 3 and 24 months after surgery (*P* > .05). Moreover, comparison of IKDC and Lysholm scores showed no statistically significant difference at each time point between the 2 groups (*P* > .05) (Table 3).

**Table 3:** Comparison of preoperative IKDC, HSS, and Lysholm scores between CKT group and traditional group (*x̄* ± *s*).

Group	CKT group	Traditional group	T value	*P* value
Preoperative IKDC	48.1 ± 16.9	47.1 ± 15.0	−0.299	.766
IKDC at 3 months after surgery	71.8 ± 9.8	68.5 ± 6.5	−1.893	.062
IKDC at 12 months after surgery	87.6 ± 6.0	87.6 ± 5.5	0.000	1.000
IKDC at 24 months after surgery	95.5 ± 3.1	92.8 ± 11.6	−1.514	.136
Preoperative HSS	57.5 ± 17.7	56.8 ± 14.3	−0.205	.838
HSS at 3 months after surgery	77.8 ± 9.4	74.5 ± 7.0	−1.856	.067
HSS at 12 months after surgery	91.9 ± 5.4	88.4 ± 4.7	−3.320	.000
HSS at 24 months after surgery	97.1 ± 2.0	96.2 ± 2.8	−1.710	.091
Preoperative Lysholm	48.7 ± 20.7	48.2 ± 19.9	−0.131	.896
Lysholm at 3 months after surgery	74.2 ± 14.9	70.3 ± 7.5	−1.563	.122
Lysholm at 12 months after surgery	90.9 ± 6.1	88.7 ± 4.7	−1.905	.060
Lysholm at 24 months after surgery	96.9 ± 3.0	96.3 ± 2.8	−1.017	.312

*Note*: HSS score at 12 months after surgery in the CKT group was higher than that in the traditional group (*P* < .05).

The results obtained using the Opti_Knee 3D motion measurement system showed that the upper and lower displacements and the anterior and posterior displacements in the 2 groups showed an upward trend, and the anterior and posterior displacements in the CKT group were significantly smaller than those in the traditional group at 24 months after surgery (*P* < .05). At the remaining time points, there was no significant difference in the upper and lower displacements and the anterior and posterior displacements between the 2 groups (*P* > .05) (Table 4).

**Table 4:** Knee kinematic analysis in the CKT group and traditional group (*x̄* ± *s*).

Period of motion	Time	Group			F value	*P* value
		Health group	CKT group	Traditional group		
Mean of maximum stride length	Preoperative	51.22 ± 3.80	47.03 ± 7.42^a^	47.02 ± 7.16^a^	H = 17.560	.000
	At 3 months after surgery	51.22 ± 3.80	50.97 ± 4.09	50.97 ± 4.09	0.59	.943
	At 12 months after surgery	51.22 ± 3.80	51.22 ± 4.41	50.95 ± 4.08	0.064	.938
	At 24 months after surgery	51.22 ± 3.80	51.12 ± 4.52	51.28 ± 4.46	0.016	.985
Mean minimum step size	Preoperative	45.02 ± 3.77	39.08 ± 4.42^a^	39.37 ± 4.13^a^	29.167	.000
	At 3 months after surgery	45.02 ± 3.77	42.95 ± 3.69	42.95 ± 3.69	4.542	.012
	At 12 months after surgery	45.02 ± 3.77	44.36 ± 4.11	43.91 ± 4.02	0.87	.422
	At 24 months after surgery	45.02 ± 3.77	44.77 ± 3.43	44.67 ± 3.70	0.107	.899
Mean step frequency	Preoperative	12.66 ± 1.20	14.11 ± 1.21^a^	14.30 ± 1.19^a^	24.617	.000
	At 3 months after surgery	12.66 ± 1.20	13.68 ± 1.03^a^	13.68 ± 1.3^a^	12.936	.000
	At 12 months after surgery	12.66 ± 1.20	12.77 ± 1.36	12.86 ± 1.25	0.286	.752
	At 24 months after surgery	12.66 ± 1.20	12.73 ± 1.17	12.77 ± 1.14	0.105	.900
Flex range of angles	Preoperative	57.42 ± 5.62	53.77 ± 7.34	53.77 ± 7.34	4.219	.17
	At 3 months after surgery	57.42 ± 5.62	52.92 ± 10.43	52.92 ± 10.43	H = 3.088	.214
	At 12 months after surgery	57.42 ± 5.62	56.18 ± 6.09	55.50 ± 4.67	1.382	.255
	At 24 months after surgery	57.42 ± 5.62	56.79 ± 7.08	56.29 ± 6.19	0.353	.703
Inside and outside turn Range of angles	Preoperative	12.64 ± 2.74	13.65 ± 3.95	13.79 ± 4.42	H = 1.890	.389
	At 3 months after surgery	12.64 ± 2.74	11.87 ± 2.89	11.87 ± 2.89	1.057	.350
	At 12 months after surgery	12.64 ± 2.74	12.54 ± 4.68	12.10 ± 3.86	0.242	.785
	At 24 months after surgery	12.64 ± 2.74	12.56 ± 3.74	11.99 ± 3.91	0.442	.644
Inner and outer rotation range of angles	Preoperative	13.71 ± 2.45	17.96 ± 5.58^a^	17.51 ± 5.33^a^	H = 16.739	.000
	At 3 months after surgery	13.71 ± 2.45	13.91 ± 5.13	14.91 ± 5.36	H = 0.816	.665
	At 12 months after surgery	13.71 ± 2.45	14.22 ± 5.76	14.72 ± 4.97	H = 0.654	.721
	At 24 months after surgery	13.71 ± 2.45	14.40 ± 4.47	14.76 ± 4.43	0.823	.441
Up and down displacement	Preoperative	1.29 ± 0.35	1.60 ± 0.36^a^	1.61 ± 0.33^a^	12.115	.000
	At 3 months after surgery	1.29 ± 0.35	1.47 ± 1.08	1.31 ± 0.67	0.73	.484
	At 12 months after surgery	1.29 ± 0.35	1.54 ± 0.35^a^	1.54 ± 0.35^a^	7.391	.001
	At 24 months after surgery	1.29 ± 0.35	1.60 ± 0.73^a^	1.55 ± 0.60^a^	3.519	.032
Internal and external displacement	Preoperative	0.84 ± 0.27	0.89 ± 0.29	0.89 ± 0.29	0.369	.692
	At 3 months after surgery	0.84 ± 0.27	0.88 ± 0.20	0.88 ± 0.20	0.379	.686
	At 12 months after surgery	0.84 ± 0.27	0.94 ± 0.31	0.93 ± 0.30	1.566	.213
	At 24 months after surgery	0.84 ± 0.27	0.91 ± 0.58	0.98 ± 0.50	H = 1.720	.423
Forward and aft displacement	Preoperative	1.47 ± 0.39	2.18 ± 1.14^a^	2.18 ± 1.14^a^	H = 13.393	.001
	At 3 months after surgery	1.47 ± 0.39	1.50 ± 0.69	1.59 ± 0.61	H = 0.676	.713
	At 12 months after surgery	1.47 ± 0.39	1.59 ± 0.70	1.79 ± 0.53	3.764	.26
	At 24 months after surgery	1.47 ± 0.39	1.66 ± 0.58*^,^†	1.84 ± 0.57*	7.214	.01

* Compared with healthy group, *P* < .05.

† Compared with the traditional group, *P* < .05.

**Figure 4.**
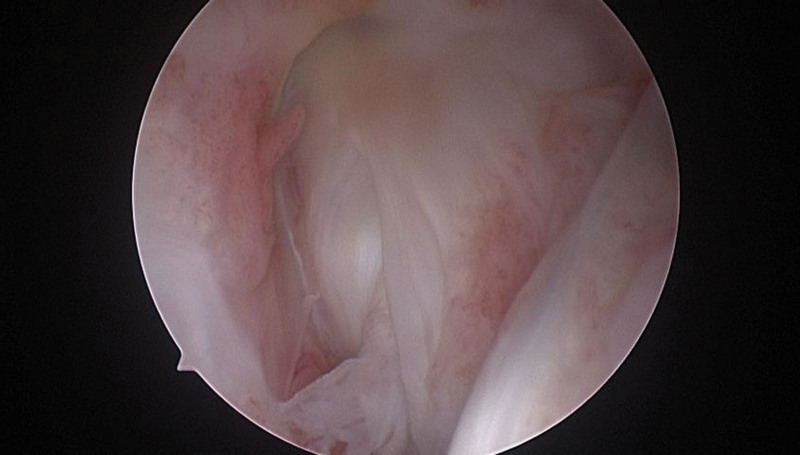
The secondary microscopic examination showed that the grafted ligaments were in the satisfactory status, the synovial membrane was well covered, and the tension was good, indicating that the grafted ligaments were of excellent MAS type. The tensioning line was located in the center with a great tension and was not broken, causing no damage or rejection of surrounding tissue.

A total of 30 of 88 patients underwent secondary arthroscopy at 12 months after surgery, including 16 patients in the CKT group and 14 patients in the traditional group. The MAS scoring criteria were used to divide the reconstructed PCL healing morphology into different grades, of which, MAS I and II were excellent (Fig. 4), while MAS III and IV were poor. Among 16 patients in the CKT group, 14 cases were excellent and 2 cases were poor by secondary knee arthroscopy, and the excellent and good rate was 87.5%. Among 14 patients in the traditional group, 11 cases were excellent and 3 cases were poor by secondary knee arthroscopy, and the excellent and good rate was 87.5%. Statistical analysis showed that the excellent and good rate in the CKT group was higher than that in the traditional group (*P* < .05) (Table 5).

**Table 5:** Comparison of excellent and good rates of MAS scores in secondary microscopy.

Group	Good quality type	Medium difference type	Total	Excellent and good rate (%)
CKT group	14	2	16	87.5
Traditional group	11	3	14	78.57
Total	25	5	30	83.33
χ^2^	0.429 .513			
*P* value				

## 4. Discussion

In 1980, Kennedy et al^[17]^ proposed the use of ligament strengthening devices to share the adverse stress to protect the tendon, while the defects of materials at that time limited its clinical application. With the development of biomaterials, FiberTape^TM^, InternalBrace^TM^, Ethibond^TM^, and other high-strength sutures exhibited satisfactory mechanical properties and biocompatibility, and they have been used for anterior cruciate ligament reduction and reconstruction.^[18–23]^ As the traditional PCL reconstruction is more likely to cause posterior tibial displacement due to graft relaxation, the proximal tibial pillow is mainly needed to prevent posterior tibial displacement postoperatively.^[24]^ Therefore, in theory, it is more effective to use CKT to prevent graft relaxation in PCL reconstruction. In our previous study, 2 2#Ethibond^TM^ wires were used to assist autologous hamstring tendon transplantation for ACL reconstruction with the internal-bracing wires woven by “triangle braiding technology,” which significantly improved the clinical efficacy and knee kinematics of patients.^[8]^ Therefore, the present study further applied CKT to PCL reconstruction, and the clinical efficacy and knee kinematics were assessed. In this study, 2 2#Ethibond^TM^ wires were used for the “triangle braiding technology” to reconstruct the internal-bracing wires through CKT-assisted PCL. The results showed that the HSS score in the CKT group was higher than that in the traditional group at 12 months after surgery, and the anterior–posterior displacement in the CKT group was lower than that in the traditional group at 24 months after surgery. The MAS score of the secondary microscopic examination at 12 months after surgery showed that the excellent and good rate in the CKT group was higher than that in the traditional group. These results confirmed that the function and stability of the knee joint in the CKT group were superior to those in the traditional group after surgery, indicating that the innovative internal-bracing wire using “triangular braiding technology” in the CKT group was beneficial to resist the adverse stretching of the graft tendon and the pulling force between the femur and the tibia on the reconstructed ligament, so as to avoid excessive stress stimulation on the reconstructed ligament during the ligament reconstruction. Preventing the reconstruction of ligament elongation is conducive to the ligament reconstruction. CKT plays a major role in the early rapid rehabilitation of the knee joint, promoting the functional rehabilitation of the knee joint and preventing joint stiffness and graft relaxation. Studies^[24–26]^ have shown that active and planned functional exercises in the early stage after anterior and posterior cruciate ligament reconstruction can effectively prevent articular cartilage and soft tissue adhesion and fibrous tissue hyperplasia, prevent muscle atrophy, enhance muscle strength, promote blood circulation of the affected limb, facilitate the subside of tissue edema and effusion, and enhance the motor coordination of the knee and surrounding tissues. Enhancement of joint proprioception function may eventually restore normal joint stability function and motor function. If the reconstructed ligament has stretch stress during the early knee rehabilitation, it may be elongated or even fractured if there is no protection before the ligament remodeling. Therefore, if the “crawling replacement” is unsuccessfully completed under a great protection after ligament reconstruction, it is difficult to obtain a promising clinical efficacy. Therefore, some researchers adopted more conservative rehabilitation measures.^[27,28]^ They demonstrated that early positive rehabilitation may lead to the risk of graft relaxation, and it is recommended to start knee bending practice at 3 to 4 weeks or even 6 weeks after surgery, while long time without knee bending practice and no weight bearing may inevitably lead to muscle atrophy around the knee and cartilage nutrition imbalance, and early postoperative conservative rehabilitation ensures the healing of the graft to a certain extent. However, in the CKT group, the innovative “triangular braiding technology” could better promote and protect the smooth completion of the crawling replacement, which is the key to greatly achieve early and medium-term function after PCL reconstruction.

To date, some studies have concentrated on reconstruction of PCL using “internal bracing reduction technique.” Zhao et al^[16]^ used the 2-0OrthoCord instrument (DePuy Mitek, Raynham, MA) to make PCL of patients with brace-reduction line reconstruction, and then, conducted a 2-year postoperative follow-up on 33 patients. The results showed that all patients were significantly improved in terms of postoperative knee function score and anteroposterior stability. During the follow-up period, no patient needed PCL revision surgery, and all patients returned to exercise. Hopper et al^[16]^ reconstructed PCL using the high-strength FiberTape suture made of long chain polymer polyethylene to make brace-reduction lines, which not only strengthened and repaired the ligament, but also retained PCL proprioception, avoided muscle atrophy of the affected limb, and was conducive to the early rehabilitation of patients. In the present study, 2 stitches were braided and then placed in the middle of the graft tendon. The tensioning thread obtained by braiding had a better strength and avoided the early rupture of the ordinary tensioning thread in the graft tendon. With placing the braided tensioning thread in the middle of the graft tendon using this method, the total diameter of the graft tendon could be prolonged to a certain extent, so that the tendon could be firmly attached to the surface of the bone tunnel. This weaving method can not only further enhance the strength and overcome the disadvantage (i.e., tensioning wire is easy to break at an early stage), but also can play the role of “internal fixation” in the tensioning wire to avoid excessive stress stimulation on the reconstructed ligament during the reconstruction of the ligament, prevent the reconstructed ligament from elongation, and facilitate the healing of the reconstructed ligament.

In summary, CKT-assisted PCL reconstruction via triangular weaving of the brace-reduction thread and placing it in the middle of the graft tendon can, to a certain extent, share the tension of the graft tendon, prevent the reconstructed ligament from elongation, be conducive to the healing of the reconstructed ligament, postoperatively combine with early rehabilitation training, and then prevent the knee joint stiffness and muscle atrophy, which is conducive to the improvement of the motor coordination and proprioceptive function of the knee joint, and finally restore the motor function of the knee joint, at last improve the postoperative recovery effect of patients, and reduce postoperative complications. It can effectively improve the recovery degree of knee function and quality of life of patients postoperatively. However, the limitations of the presented study should be pointed out. For example, Riccardo et al^[29]^ followed 643 participants for more than 1 year to report the effects and complications of the rachion-preserving techniques in Anterior cruciate ligament reconstruction. This indicates that our team’s follow-up study should expand the sample size and carry out a multi-center, prospective study to further verify the effect of arthroscopic internal reduction technology assisted PCL reconstruction.

## Acknowledgments

Thanks for Professor Guo Quanyi of the Department of Orthopedics of the Chinese People’s Liberation Army General Hospital for his guidance and support in this study.

## Author contributions

**Conceptualization:** Bohan Xiong.

**Data curation:** Bohan Xiong, Guoliang Wang, Yanlin Li.

**Formal analysis:** Yanlin Li.

**Investigation:** Bing Xie, Xianguang Yang.

**Resources:** Bohan Xiong, Bing Xie.

**Validation:** Jinrui Liu.

**Visualization:** Xianguang Yang, Ziming Gu.

**Writing – original draft:** Bohan Xiong.

**Writing – review & editing:** Yang Yu.
